# In favour of tobacco control? Former smokers’ support for tobacco policies

**DOI:** 10.1177/1455072519853914

**Published:** 2019-07-04

**Authors:** Tove Sohlberg

**Affiliations:** Stockholm University, Sweden

**Keywords:** former smokers, nicotine users, policy, snus, Sweden, tobacco control

## Abstract

**Background:**

Tobacco control (TC) in Sweden is being continuously strengthened.

**Aims:**

The study aimed to examine attitudes towards different TC policies among former smokers, the difference between nicotine-free former smokers and those who use nicotine in the form of snus or nicotine replacement therapies (NRTs), and whether different TC strategies tend to become more acceptable over time.

**Methods:**

Respondents are part of a seven-year follow-up of former smokers in Sweden. Initially, 1400 respondents were contacted regarding participation and 705 answered a survey (response rate 50%). The present study used cross-sectional data on attitudes towards different TC policies and respondent’s level of support were measured on a 4-point scale. Analyses consist of percentage distribution of level of agreement, in total and between nicotine-free individuals and users of nicotine in the form of snus or NRTs, as well as logistic regressions in order to predict the odds for supporting the different policies. In addition, a percentage distribution of support for different policies introduced during different time-periods is shown.

**Results:**

There is an overall support for smoke-free environments. Nicotine users are, however, overall slightly more opposed, especially to policies aiming at denormalising smoking. Public support is important for successful implementation but resistance can pass, and interventions tend to become more acceptable over time.

**Conclusion:**

While smoke-free indoor environments can be justified by scientific evidence of harm to others, bans against smoking outdoors might be experienced as intrusive. Policies need to rest on scientific arguments and be seen as appropriate actions, underlining the importance of information for successful implementations.

With an internationally low smoking prevalence, the Swedish regime of tobacco regulation might be seen as a shining example for other countries. Only about 11% of the Swedish population (Statistics Sweden [SCB], 2016/2017) are daily smokers, the lowest rate of all the Nordic countries and even in the entire European Union (EU) (Eurostat, 2016). However, when including the use of snus (moist tobacco to put under the lip – which, in the EU, is only allowed to be sold in Sweden) total tobacco use adds up to about 22.5% (SCB, 2016/2017), which is more on a par with other European countries. Yet, in spite of the low smoking prevalence, about 12,000 people in Sweden die each year due to the habit and almost 100,000 are annually (2010–2012) afflicted by smoking-related diseases (Swedish National Board of Health and Welfare, 2014). In order to mitigate this public health issue, a number of tobacco control policies have been introduced – some have already been implemented and others are soon to come into force.

When it comes to public opinion on tobacco control, certain municipalities have conducted surveys, but as national polls on the matter are rarely carried out, gaining an overview of public attitudes is quite hard to achieve. There are some data to support the claim that smokers in Sweden have a more negative attitude than non-smokers towards certain tobacco control policies (Swedish Cancer Society, 2016; as well as a number of international studies: Ashley et al., 2000; Lazarus et al., 2009; Lund, 2016). Former smokers have also been found to be generally more supportive of tobacco control policies than current smokers (Schumann et al., 2006). Quite recent opinion polls show strong support for smoking bans in public spaces such as outdoor seating areas in restaurants, bus and train platforms, and in playgrounds (Arkhede, Bergström, & Ohlsson, 2016; Swedish Cancer Society, 2016), so there is apparent support for a strengthening of controls.

In a democracy the public must be kept informed and public policies should be approved by the people, since the people are the ultimate source of power (American Historical Association, 2018). However, smoking in Sweden is declining and, as stated in a study from Norway, smokers are becoming a minority group with a diminishing public voice, meaning that public support is almost equivalent with non-smokers opinions (Lund, 2016). But as new categories with different experiences of tobacco use or of other forms of nicotine emerge, it is of interest to take the perspective of these groups into account. For example, former smokers constitute a group in between the current smokers and the never smokers, having experience both of being smokers and of becoming non-smokers.

So, what about these former smokers? Do they empathise with the non-smoking public overall, or are their opinions more in line with the opinions of smokers, having been smokers themselves? And is there any difference in attitudes between former, now nicotine-free smokers and those still using nicotine in the form of Swedish snus or nicotine replacement therapies (NRTs) such as plasters and chewing gum? It is important to know how the public, including those who themselves have experience of smoking, perceive these policies. Current data give a great opportunity to investigate this from a former smoker perspective.

The main focus of the present study was to examine attitudes towards both longstanding and recently adopted tobacco control policies among individuals in Sweden who have once been daily smokers. More specific questions are: Is there any difference between those who have become wholly nicotine free and those who have quit daily smoking but use nicotine in the form of snus or NRTs? And do different tobacco control strategies tend to become more acceptable over time for these former smokers?

## The emergence and development of Swedish tobacco policy

The view of smoking as a problem has changed over time, as have the strategies designed to solve it. First, the idea of information being the key to a healthier lifestyle without tobacco dates back to the beginning of the 1900s, and up until the 1960s tobacco control in Sweden was characterised by a consensus-driven belief in the power of information directed towards active smokers. Thereafter, warning labels on cigarette packages were introduced during the 1970s, followed by restrictive legislation concerning advertising in the media. When international studies in the 1980s showed a causal effect of passive smoking on health (Hirayama, 1981; Trichopoulos, Kalandidi, Sparros, & Macmahon, 1981), a transition took place from the rational choice perspective to the perspective of harm to others, which might be one of the explanations behind the consensus-oriented tobacco controls (Cisneros Örnberg & Sohlberg, 2012).

The Swedish Tobacco Act (1993:581) came into force in 1993, when smoking on public transport and in public indoor places was banned, as well as smoking in workplaces, except in specially designated spaces. However, restaurants and bars were not at that time included in the smoke-free working environment category, so there smoking was not banned until 2005. Further, an age limit of 18 years for purchase of tobacco was introduced. At the time of this study, the Act laid down rules on smoke-free environments, marketing and warning texts, but over time added further provisions.

In 2016 an amendment to the Tobacco Act (2016:353) was added, ruling that packaging of tobacco products must be accompanied by both warning texts and illustrations. The question of plain packaging was also raised (already implemented in, e.g., Australia), but was found to contravene the Freedom of the Press Act. An implementation would therefore require an amendment to the Constitution, something not found to be relevant (SOU, 2016:58). In order to further implement guidelines and protocols of the World Health Organization (WHO) Framework Convention on Tobacco Control, the EU Tobacco Products Directive (see section on WHO and EU input below), as well as their own national Alcohol, Narcotics, Doping, and Tobacco (ANDT) policies, the government submitted a bill in January 2018 suggesting *New rules on tobacco and similar products* (Council on Legislation [Lagrådsremiss], 2018). One of the matters reviewed was the possibility of implementing a display ban on tobacco products; something that our Scandinavian neighbours Norway, Iceland and Finland already have in place. However, this was considered an unnecessary measure (Council on Legislation [Lagrådsremiss], 2018) and it was decided that in Sweden marketing in the form of displaying tobacco products and pricelists should be allowed if not intrusive, outreaching or urging to use tobacco.

The new law (with legislative amendments adopted in December 2018 to come into force in July 2019) further includes an extended smoking ban in certain outdoor public spaces such as outdoor seating areas at restaurants/bars, entrances to smoke-free buildings, outdoor platforms of buses and trains, sports arenas, and playgrounds, also banning the use of e-cigarettes, with or without nicotine (Lagrådsremiss, 2018). Snus, being a tobacco product, is also covered in this new law, focusing, however, on marketing and authorisation to sell the product.

The official Swedish standpoint on snus is that it is a less harmful alternative to smoking and therefore should be regarded as a potential means of becoming smoke free (Cisneros Örnberg, 2013). This same argument has been used by the government in appeals to the EU concerning the export ban on Swedish snus to the EU market (C-210/03 and C-434/02), and by the industry which wants to keep and expand the market for snus (C-151/17).

### WHO and EU input

Two actors with great influence on Sweden’s tobacco control are the World Health Organization (WHO) and the European Union (EU). The WHO Framework Convention on Tobacco Control (WHO FCTC) came into force in 2003 and Sweden signed the treaty in 2005 (WHO FCTC, 2009). Since implementation of the WHO FCTC is a legal commitment, Sweden was required to adjust its national tobacco law in line with it. Moreover, as an EU member state since 1995, various EU directives concerning tobacco control have gradually been incorporated into Swedish law. However, smoking in Sweden had already started to decline during the 1980s and the current low prevalence is therefore not a direct consequence of the country’s adaption to WHO and EU tobacco control strategies.

## Successful implementations?

A successful, frictionless implementation of tobacco control policies comes from a combination of public support, the manner of framing, and how they are introduced. In Sweden, tobacco taxation is enshrined in a separate law (1961:34) dating back to the 1960s, in spite of which demands for an active price policy were not raised until the 1990s. The EU introduced common minimum tax levels in 1993, which Sweden put in place in 1997, raising the price of cigarettes. But this provoked a severe backlash consisting not only of an increase in cigarette smuggling and cross-border shopping, but of deep opposition to more expensive tobacco products. As a result, the Swedish parliament was moved to lower the taxes to previous levels. This experience emphasises the need for public support for successful implementation of control policies.

Not all policies are laid down in laws and regulations, however; some are actually voluntary. For example, Sweden’s municipalities are encouraged to work not only for tobacco-free workplaces, but also for tobacco-free working hours. Thus, a majority of Sweden’s municipalities (201 of 290) have launched smoke-free working days (Tobaksfakta, 2014). In line with this, a few municipalities (16 of 209) also include snus in this ban (Thorin & Thorsson, 2016). Of course, the use of cigarettes during breaks along with the use of snus is hard to control (especially the latter since they are easily slipped in/placed under the lip), as well as the fact that such violations of policy are not followed up with legal action. Overall, however, interventions perceived as personally intrusive have proven to gain less support, even where tolerance is stronger for tobacco control than for interventions for alcohol use, diet or physical activity (Diepeveen, Ling, Suhrcke, Roland, & Marteau, 2013).

The eventual success of tobacco control policies is also a matter of framing. As smoking is framed as both a personal and an environmental health issue, existing and proposed policies directed at solving these problems must be seen as appropriate (Björkdahl, 2008), which explains why successful implementation of new policies also depends on how they are introduced to the public. The absence of a proper introduction was actually why the Public Health Agency of Sweden was in agreement with the Council on Legislation (Lagrådsremiss, 2018) regarding non-implementation of a display ban. Moreover, support for different policies in the field of tobacco control has been shown to be related to the stage of implementation, where interventions became more acceptable once they had been introduced (Diepeveen et al., 2013).

All in all, these regulations of course aim to reduce smoking through a combination of controlling availability and demand. In addition, passive smoking prevention strategies are considered to be an effective factor for protecting others from exposure to harm in social settings, as well as being supportive of those desiring to become smoke free (The Public Health Agency of Sweden, 2014). The WHO expects that extensive smoke-free laws will reduce the status of smoking, especially among teenagers (WHO, 2007). Hence, a reduction of social exposure to smoking is an important measure in denormalising smoking (Collins & Procter, 2011; SOU, 2016:14, p. 247) – a change in social norms that should lead to a reduction of cigarette consumption (Alamar & Glantz, 2006).

## Smoking cessation strategies

In the 1980s in Sweden there were about as many women as men smoking on a daily basis. From then on, there has been an overall decrease in smoking habits, partly due to a decline in smoking initiation but also to successful smoking cessations. The overall aim of Sweden’s tobacco control strategies is to reduce smoking prevalence and it is likely that smoke-free policies do influence willingness to quit smoking. Both restrictions and interventions have become more multifaceted over time, the latter including medicinal aid (e.g., NRTs), as well as other professional treatments. Even so, a quite common way for Swedes to become smoke free is with the assistance of snus (about 26% of the Swedish men who use snus are former smokers: The Public Health Agency of Sweden, 2014) which is not, however, recommended by the EU as a means of becoming smoke free (European Commission, 2008).

So, among successful quitters who make use of an aid to become smoke free, snus is by far the preferred product (Lund, Scheffels, & McNeill, 2011; Ramström & Foulds, 2006; Scheffels, Lund, & McNeill, 2012), and for every individual who goes from snus to smoking there are about four individuals who go from smoking to snus (Swedish National Board of Health and Welfare, 2005). There are, however, distinct gender differences, and findings from Norway show that women tend to prefer NRTs such as nicotine chewing gums and plasters to using snus (Scheffels et al., 2012), since snus as a means of becoming smoke free may easily result in continued use (Lund et al., 2011; Scheffels et al., 2012; Sohlberg, 2015).

The general aim of the present study was thus to examine the attitudes towards different tobacco control policies in Sweden among former smokers. Are they supportive overall? Is there any difference between nicotine-free former smokers and users of nicotine in the form of snus or NRTs? Do different tobacco control strategies tend to become more acceptable over time for these former smokers?

## Data and method

### Data

The respondents in this study were originally recruited from the so-called Monitor Project, a running monthly survey directed to a representative sample of 1500 respondents aged 16–84 years (in total, 18,000 per year). The Centre for Social Research on Alcohol and Drugs (SORAD, now within the Department of Public Health Sciences at Stockholm University) conducted the project from 2000 to 2012. During the period October 2009 to May 2010 a comprehensive sample of former smokers (*n* = 1882) was recruited for a study about smoking cessation, where the response rate was over 89% (*n* = 1683). These respondents are now part of a seven-year follow-up with the overall aim of revealing factors affecting long-term smoking cessation on an individual level and, as investigated in this article, attitudes to different policies. Both studies were approved by the Regional Ethical Review Board in Stockholm (2009/2102-31/5; 2017/561-31/5).

In total, 1400 respondents were contacted regarding participation and asked to answer a web-based survey that ran from August 2017 to February 2018. Of these, 283 individuals could not be found for various reasons, such as unknown address, moved abroad, deceased. The response rate was about 50% (*n* = 705), but a non-response analysis showed no significant differences between participants and non-participants in such sample characteristics as gender, age, education, economic status and time of cessation.

The respondents are all now smoke free on a daily basis, although a smaller share is still using nicotine, either in the form of snus or NRTs. Those respondents were excluded who had started to smoke again (*n* = 8), smoked only occasionally (*n* = 21), or smoked occasionally but used snus or NRTs daily (*n* = 13). Thus, included in the data were former smokers (*n* = 609) who still are both smoke and nicotine free and those who stated regular use of snus or NRTs (*n* = 54).

### Variables

In order to give a background description of the sample, the respondents’ *gender*, *current age*, *time of cessation* and *current use of snus or NRTs* are shown in Table 1. Respondents were asked to state their gender as female, male or other. Only one respondent stated other and was left out of the analysis. Current age was calculated from the question “which year were you born?” and ranged from 36 to 89 years. Current use of nicotine was measured through a two-step process. First, respondents were asked whether they had made use of any nicotine product in their smoking cessation process, and if so “what kind of nicotine product did you mainly use?” where the respondent could tick a box with alternatives such as snus and different forms of NRTs such as chewing gum, plasters, oral spray or tablets. Second, respondents were asked to state time of usage where one alternative was “still using” (snus or NRTs). These two alternatives for nicotine use were merged into one single variable and constitute the base for being a “nicotine user”. *Time of cessation* was measured using “You have been smoke free since (year)…” and thereafter divided into four different decades (1970s, 1980s, 1990s and 2000s) based on the time of implementation of different tobacco control strategies.

**Table 1.** Descriptive background of the sample.

*Gender (%) (*n* = 662)	
Women	54.4
Men	45.4
**Current age** (mean) (*n* = 663)	66.9 (std 10.8)
**Time of cessation** (*n* = 663)	
1970s	21.4
1980s	21.7
1990s	25.5
2000–	31.4
**Current use of snus or NRTs (%)** (*n* = 54; snus *n* = 49, NRTs *n* = 5)	8.1

NRTs = nicotine replacement therapies; std = standard deviation.

*One respondent stated the alternative “other” which is why gender figures do not add up to 100%.

Of special interest for the present study is an inventory, included in the survey, regarding attitudes towards different laws and regulations aimed at restricting smoking and other nicotine use in the society. At the time of the survey some of these policies had been proposed by the government, but not yet adopted (scheduled for July 2019), and this is what the respondents have taken a position on. The inventory consists of 18 bans and proposed restrictions where respondents could mark on a four-grade scale whether they *Do not support* (1 = no support) or *Support* (4 = full support). These bans and restrictions were chosen for their direct impact on the individual, as related to the sales of cigarettes.

The inventory covers all such policies from:

- the Tobacco Act 1993 (bans against smoking in public buildings, on public transport, in workplaces),
- sharpening of policies over time (bans against smoking in restaurants/bars, warning labels in the form of texts or pictures on cigarette packages),
- the then proposed and soon to be adopted policies (bans against smoking at entrances with public access, at public transport platforms/bus stops/taxi zones outdoors, at outdoor seating areas at restaurants/bars, at sports arenas, at playgrounds, which also apply to e-cigarette use, along with bans against flavours),
- policies not adopted in the new Law on Tobacco and Similar Products (plain packaging and display ban not finally proposed on the grounds of contravening other laws, tax increases),
- voluntary policies relating to tobacco-free workdays (total smoke- and snus-free workdays).

Not included are such restrictions as product requirements, reporting obligation and supervision, since these do not affect the everyday life of the individual. Nor is marketing included since the regulations are hard to grasp for non-professionals. The items included in the inventory are shown in Table 2.

**Table 2.** Attitudes towards existing and proposed tobacco control policies. Percentages supporting (to a great extent/totally) each policy. Logistic regressions on nicotine-free vs. nicotine-user support for each policy, controlling for gender and age. Odds ratios (CI 95%).

	Nicotine free (*n* = 609)	Nicotine users (*n* = 54)	Total (*n* = 663)	Sig. difference	Logistic regression
	%	%	%	*p*-value	*OR* (ref. category = nicotine users)
**Bans against smoking…**					
in public buildings	96.2	92.6	95.9	.206	ns
at entrances where the public has access	93.2	81.5	92.2	.002	0.40** (0.15–0.69)
on public transport	97.5	100.0	97.7	.240	ns
at platforms, bus stops, taxi zones outdoors	83.4	72.2	82.5	.038	ns
in restaurants, bars	95.5	96.2	95.6	.804	ns
at outdoor seating areas at restaurants/bars	81.2	69.8	80.3	.046	0.51* (0.29–0.99)
at sport arenas	91.4	75.9	90.1	.000	0.32** (0.15–0.59)
at playgrounds	94.5	83.7	93.9	.029	0.44* (0.17–0.93)
in workplaces	87.5	74.1	86.3	.006	0.59** (0.21–0.79)
.. and a total smoke-free workday	79.4	63.0	78.1	.005	0.60** (0.25–0.79)
.. and a total “snus”-free workday	56.6	5.7	52.4	.000	0.06*** (0.01–0.15)
That the above bans also apply to e-cigarette use	76.5	56.6	74.9	.001	0.43** (0.23–0.71)
**Information**					
Warning labels in form of texts on cigarette packages	89.9	90.6	90.0	.886	ns
Warning labels in form of pictures on cigarette packages	84.6	70.4	83.4	.007	0.45* (0.23–0.81)
**Sales of cigarettes**					
Plain packaging	55.3	39.6	54.0	.028	0.55* (0.23–0.94)
Display ban	74.0	48.1	71.8	.000	0.30*** (0.18–0.57)
Tax increase on cigarettes	77.0	50.9	74.9	.000	0.28*** (0.18–0.55)
Ban against flavours such as, e.g., mint	61.4	28.3	58.7	.000	0.27*** (0.13–0.46)

*p < 0.05. **p < 0.01. ***p < 0.001.

### Analysis

Initially, in order to illustrate level of agreement, the scale for each policy was dichotomised into *No support* (not at all/to some extent) and *Support* (to a great extent/totally).

Since the data also include a number of respondents who are smoke free, although not nicotine free, the total respondent group was divided into the *Nicotine free* and the *Nicotine users*, in order to compare potential differences in attitudes towards tobacco control policies connected to nicotine status. Hence, the attitudes of the users of snus or NRTs constitute a comparative group in the percentage distribution of agreement. The differences in relation to nicotine-free respondents are assessed using chi^2^. In order to predict the odds for supporting the different policies, logistic regressions (statistical program SPSS 23) were performed. The dependent variables were no support/support (0–1) for each policy and the independent variable was nicotine free/nicotine user (0–1), controlling for gender and age.

Moreover, the items in the inventory were summarised to form three categories: Policies implemented during the 1990s, Policies implemented during the 2000s, and Policies proposed to come into force during the 2000s. The construction of these indices was carried out on the basis of when each policy was introduced. Each index was then divided into No support or Support. Hence, Table 3 shows the percentage distribution of level of support towards existing as well as proposed tobacco control policies introduced during different time-periods.

**Table 3.** Attitudes towards existing and proposed tobacco control policies during different time-periods. Percentages supporting (to a great extent/totally) each policy.

	**Total**
Policies implemented during the 1990s (n = 645)	98.3
Policies implemented during the 2000s (n = 634)	95.9
Policies proposed to enter into force during the 2000s* (n = 613)	95.3

*at the time of the survey several policies had been proposed, but not yet implemented.

Not included in these categories is a total smoke- and snus-free workday, since this is a voluntary policy, and tax increases, since these have taken place on different occasions over time. The possibility of implementing plain packaging and a display ban was examined, but on the grounds of conflicting legislation was not even proposed. Therefore, these two policies are not included either.

## Results

### Background

Basic sample descriptives and time of cessation are shown in Table 1. As indicated in Table 1 there are more women than men in the sample, and a mean age of about 67 years. About 40% of the respondents quit smoking as long ago as 30–45 years. A quarter quit smoking during the 1990s, which is about 20–30 years ago. The remaining third became smoke free early in this century (the 21st). Only about 8% (*n* = 54) are still currently using nicotine. However, almost 17% made use of snus while quitting (not shown in the Table) and of those, almost 51% are still using this form of nicotine. Those former smokers who use snus or NRTs are quite recent quitters with about 54% becoming smoke free during the 2000s.

### Attitudes towards tobacco control policies

The analysis of attitudes to existing tobacco control policies in Sweden, but also, at the time of the study, proposed tobacco controls (Table 2), reveals overall major support from former smokers in total. As shown, there is very strong support (from around 82% to over 90%) for bans aimed at protecting non-smoking others from harm. However, there is slightly lower reported support for a ban against smoking at restaurant/pub outdoor seating areas, for a total smoke-free workday, and for letting these bans against smoking also include e-cigarette use. Of the studied policies, the snus-free workday was the least supported. Reactions to the idea of plain packaging and a ban against flavours in tobacco point in the same direction. On the other hand, information on packages in the form of warning texts and pictures have quite strong support.

Not surprisingly, there are significant differences between nicotine-free persons and those who use nicotine in some form. Even though a majority of the nicotine users support bans against smoking they were more in opposition than the nicotine-free respondents were. What stands out is that where bans against smoking include e-cigarette use, only just about half of nicotine users support this. Not too surprisingly either, the nicotine users show extremely little support for a total snus-free workday. Information in the form of pictures on the cigarette packages induces quite strong support, even though fewer nicotine users than nicotine-free respondents support this. As stated above, relatively few in the total sample support plain packaging and a ban against flavours in tobacco, while a majority of the nicotine users were opposed both to this and to a display ban. However, just about half of the nicotine users actually supported a tax increase on cigarettes. Further, a logistic regression performed in order to investigate the influence of nicotine status on support for each policy showed that nicotine status is highly skewed, with very few nicotine users. The results should therefore be viewed with some caution. However, the odds ratio supports the percentage distribution, with lower support among nicotine users, and taken together gives quite a clear indication.

At a glance, it seems that there is stronger support for policies already implemented, for example during the 1990s. This is examined in Table 3. Overall, a clear majority of former smokers support both the already implemented and the proposed (but not yet implemented) policies. Even though the difference in support between the decades is very small, it is significant – indicating that support increases somewhat over time. Hence, more respondents (about 98%) support the policies implemented during the 1990s than the policies adopted during the 2000s (about 95%) even though they mostly cover bans intended to create more smoke-free environments.

## Discussion

This study aimed to examine attitudes towards implemented and, at the time of the study, proposed tobacco control policies among Swedish former smokers. Previous studies from, e.g., Germany (Schumann et.al., 2006), and an ESTA project covering Germany, Greece, Poland, Sweden and UK (Lazuras et.al., 2009) have found that former smokers are more supportive of tobacco control measures than current smokers.

The main findings are that there was major support for all policies in total, and especially for those policies aiming to protect others from the effects of passive smoking. Even if the support was overwhelmingly wide and in line with general public support, there was slightly less support for the proposed bans than for those that had already been implemented, illustrated by categorising the bans into different time-periods. Moreover, a distinct difference in attitudes emerged between nicotine-free individuals and those still using nicotine in the form of snus or NRTs, where nicotine users were significantly less supportive of restrictions. Most apparent was the difference in attitudes towards the proposal that e-cigarette use should not be allowed in smoke-free areas.

Sweden’s low smoking prevalence suggests successful tobacco control, or at least a successful control of smoking. Actually, the implemented and the proposed policies included in this study consistently relate to regulation of (cigarette and e-cigarette) smoking, but not tobacco regulation as such, and even though snus is included in the new law concerning marketing, there is no such thing as a wholly tobacco-free environment. The government supports the goal of “Tobacco Endgame 2025 – A smoke-free Sweden”, run by a number of organisations working with tobacco-use prevention (Swedish Government, 2015), but this very heading emphasises the main point: that tobacco free is equated to smoke free in a way that is unclear, and that the mixing of these two concepts complicates the debate on harm reduction.

The recent legislation is a logical progression from existing policy with focus on strengthening the protection of others from being involuntarily exposed to tobacco smoke. These environmental smoking prevention strategies are not new, however, dating back to the 1980s when the Tobacco Committee and the National Board of Health and Welfare, in consequence of the evidence of a link between smoking and harm to non-smoking others, began to prepare guidelines for creating smoke-free environments (SOU, 1981:18). Hence, the country was already in the process of tobacco-related policy making when the WHO FCTC and the EU tobacco control strategies were adopted, possibly explaining part of the public consensus regarding revisions and strengthening of the Tobacco Act.

Apart from protecting others from harm, the reduction of social exposure to smoking also refers to a denormalisation of smoking, in order to bring about a change in attitudes on a cultural level, and this is why e-cigarette use is included in the new Law on Tobacco and Similar Products (Lagrådsremiss, 2018). However, e-cigarette use is still quite uncommon in Sweden (only about 8% state ever having tested it: Eurobarometer 458), so it does not constitute a problem for the public to the same extent as regular cigarettes. This may explain the slightly lower share in total support for this (as shown in Table 2).

Significant differences in attitude towards tobacco policies were found between those who are totally nicotine free and those who still use nicotine in the form of snus or NRTs, where the nicotine users overall seem to have a slightly more liberal attitude. However, more than half of all nicotine users in the present study quit smoking during the 2000s and might still identify with smokers more than non-smokers. The totally smoke- and snus-free workday is neither implemented nor a proposed policy but is suggested as a voluntary measure. However, the nicotine users in particular were greatly opposed to a snus-free workday. This is of course not really breaking news since most of them use snus on a daily basis, but it may also be that such a policy feels quite intrusive of personal space, since snus is placed under the lip. Why is this to be controlled? And by whom?

High discrepancies were also found for smoking at roofed stands for transport and outdoor seating areas, that the bans aiming to create smoke-free environments also apply to e-cigarette use, warning labels in the form of pictures on cigarette packages, plain packaging, a display ban, tax increases on cigarettes, and flavouring, while attitudes towards other policies were more equivalent. The negative effects on health due to passive smoking indoors has been known since the 1980s, but it is a challenge to make bans like smoking at outdoor platforms/bus stops and at outdoor seating defensible without basing such bans on scientific arguments concerning health risks to others (Sohlberg, 2016). Also, it has been pointed out that convincing smokers to regulate their behaviour based on research pertaining to health consequences seems easier than attempts to convince them based on denormalisation approaches (Lund, 2016). This is when the role-model argument becomes relevant: reducing opportunities for people to observe smoking in public spaces should lead to smoking no longer being perceived as a normal behaviour (Collins & Procter, 2011). Relocating cigarette smokers as well as e-cigarette users to marginal places fills the same function.

As bans on indoor smoking are an integrated part of Swedish tobacco control it is a logical progression to extend these bans to also include outdoor smoking. However, such bans refer more to denormalisation than protecting others from harm, and former smokers in total also give more support to policies aiming at protecting others than to policies aiming to denormalise smoking, and this is especially so of former smokers who still use nicotine.

An inquiry as early as 1918 into the growing use of tobacco concluded that information was the key to a healthy lifestyle (Tobaksfakta, 2011). So, Sweden has a long history of promoting the role of information, which still constitutes an important part of the country’s tobacco control strategy. One lesson learned from the failure of the tax increase in 1997 was the need for public support for successful implementation, and that this support can be obtained through information campaigns. For example, the policies that entered into force in 2019 had been introduced to the public already throughout 2018 via such media as the daily press and the internet, and, as the findings of the present study show, Swedish tobacco policies gained overall support from former smokers.

Hence, implementation is a question of how to introduce new polices to gain maximum support. Even if initial support seems important it has been shown that interventions tend to become more accepted once they have been implemented (Diepeveen et al., 2013). An example from the Swedish context is the criticism of the smoking ban in restaurants that was quite strong before the ban came into force in 2005 (especially by the restaurant industry itself) but the ban was positively met by the general population shortly after. This supports findings from previous studies that immediate resistance can pass and turn into acceptance over time (see for example Heloma & Jaakkola, 2003; Hyland et al., 2009; Thomson, Wilson, & Edwards, 2009). The results of the present study are in line with this experience: more respondents supported policies implemented during the 1990s than those coming into force during the 2000s, even though the overall main focus is smoke-free environments. It has been thought that smoking bans helping to create smoke-free environments contribute to a denormalisation of smoking, in turn leading to further support for such restrictions (Collins & Procter, 2011). However, policies must be seen as appropriate actions for solving the problem (Björkdahl, 2008) and need to rest on scientific arguments. In fact, there is a risk of tobacco control advocates being branded as extremists whose agenda abandons all proportionality (Chapman, 2007), with such policies as smoke- and snus-free workdays (with its apparent difficulty of securing compliance). When employers introduce totally tobacco-free workdays it is, however, not only to restrict behaviour but also to improve the health of employees.

### Limitations and strengths of the study

The results of this investigation must be considered in light of certain limitations. First, the Monitor data on which the present study are based are thought to constitute a representative sample of the Swedish population. However, the market research company simply interviewed a total of 1500 individuals each month and previous respondents who could not be reached were replaced with others. The missing data have increased over time and were as large as 60% in 2010 (Ramstedt, Lindell, & Raninen, 2013). The first study of former smokers (from 2010) consisted of 1683 respondents. This seven-year follow-up contacted 1400 regarding participation, finally ending up with 705 (response rate around 50%). Altogether, this raises the issue of representativity. Second, over 40% quit smoking during the 1970s and 1980s, and therefore should in principle be considered as non-smokers, having abandoned the habit before most tobacco control policies were even proposed. The sharpening of Swedish tobacco policy over time probably has led to a more negative attitude toward smoking, an attitude they have had a long time to make their own. Third, the mean age of 67 years means that these results reflect the attitudes of an older generation and would maybe be different if from a younger sample. Fourth, current use of snus or NRTs is merged into one variable, nicotine users, since there are only five respondents using NRTs daily. It is possible these two kinds of users may differ from each other, but the probability is that the impact of this small sample of NRT users is negligible. Lastly, time of cessation dates back in time and there is most certainly a recall effect, but previous studies have concluded that recall of smoking status is usually accurate (Bernaards, Twisk, Snel, van Mechelen, & Kemper, 2001; Kenkel, Lillard, & Mathios, 2003).

Keeping this in mind, the present data, where over 89% of the recruited former smokers participated in the study in 2010, are rather unique in a Swedish context. Even more so is this follow-up where almost half were eager to participate as long as seven years later. This indicates not only a commitment to contribute to tobacco research, but an interest in the topic itself. It is therefore concluded that their answers are probably thoughtful.

## Conclusion

The overall aim of Sweden’s tobacco control policies is to reduce smoking prevalence. Even though a new law has been adopted recently, and several of the WHO FCTC and the EU tobacco policies have already been successfully implemented, Sweden is not the most restrictive country. Not all proposed actions have been taken, such as plain packaging or a display ban. Moreover, Sweden is the only country in the EU exempt from the sales ban on tobacco for oral use (not intended to be smoked or chewed) (Cisneros Örnberg, 2013) – that is, snus. In any event, considering the already very low smoking prevalence in Sweden, it seems that the goal of “Tobacco Endgame 2025 – A smoke-free Sweden” might indeed be achievable. However, this is only if relating to the latter part, namely “a *smoke-free* Sweden”. If instead relating to “*Tobacco Endgame*”, the great number of snus users has to be taken into account. Not only do about 13% of Swedish people (around 21.5% of men, 4% of women) use snus on a daily basis (SCB, 2016/2017), it is also very common to use snus as a means of becoming smoke free. Public support for eventual restrictive policies concerning snus and its use is yet to be seen, and is an important topic for future research.
